# Overexpression of corticotropin-releasing factor in intestinal mucosal eosinophils is associated with clinical severity in Diarrhea-Predominant Irritable Bowel Syndrome

**DOI:** 10.1038/s41598-020-77176-x

**Published:** 2020-11-26

**Authors:** Eloísa Salvo-Romero, Cristina Martínez, Beatriz Lobo, Bruno K. Rodiño-Janeiro, Marc Pigrau, Alejandro D. Sánchez-Chardi, Ana M. González-Castro, Marina Fortea, Cristina Pardo-Camacho, Adoración Nieto, Elba Expósito, Danila Guagnozzi, Amanda Rodríguez-Urrutia, Inés de Torres, Ricard Farré, Fernando Azpiroz, Carmen Alonso-Cotoner, Javier Santos, María Vicario

**Affiliations:** 1 Laboratory of Translational Mucosal Immunology, Digestive System Research Unit, Vall D’Hebron Institut de Recerca, Department of Gastroenterology, Hospital Universitari Vall D’Hebron, Universitat Autònoma de Barcelona, Paseo Vall d’Hebron, 119-129 Barcelona, Spain; 2 Laboratory of Neuro-Immuno-Gastroenterology, Digestive System Research Unit, Vall D’Hebron Institut de Recerca, Department of Gastroenterology, Hospital Universitari Vall D’Hebrón, Universitat Autònoma de Barcelona, Barcelona, Spain; 3 Servei de Microscopia, Universitat Autònoma de Barcelona, Barcelona, Spain; 4 Department of Psychiatry, Hospital Universitari Vall D’Hebrón, Universitat Autònoma de Barcelona, Barcelona, Spain; 5 Centro de Investigación Biomédica en Red de Salud Mental (CIBERSAM), Madrid, Spain; 6 Department of Pathology, Hospital Universitari Vall D’Hebrón, Universitat Autònoma de Barcelona, Barcelona, Spain; 7 Translational Research Center for Gastrointestinal Disorders (TARGID) KU, Leuven, Belgium; 8 Centro de Investigación Biomédica en Red de Enfermedades Hepáticas y Digestivas (CIBEREHD), Madrid, Spain; 9 Lleida Institute for Biomedical Research, Lleida, Spain; 10 Present Address: Department of Gastrointestinal Health, Société Des Produits Nestlé S.A, Nestlé Research, Vers-chez-les-Blanc, 1000 Lausanne 26, Switzerland

**Keywords:** Irritable bowel syndrome, Mucosal immunology

## Abstract

Corticotropin-releasing factor (CRF) has been identified in intestinal mucosal eosinophils and associated with psychological stress and gut dysfunction. Irritable bowel syndrome (IBS) is commonly characterized by altered intestinal motility, immune activation, and increased gut barrier permeability along with heightened susceptibility to psychosocial stress. Despite intensive research, the role of mucosal eosinophils in stress-associated gut dysfunction remains uncertain. In this study, we evaluated eosinophil activation profile and CRF content in the jejunal mucosa of diarrhea-predominant IBS (IBS-D) and healthy controls (HC) by gene/protein expression and transmission electron microscopy. We also explored the association between intestinal eosinophil CRF and chronic stress, and the potential mechanisms underlying the stress response by assessing eosinophil response to neuropeptides. We found that mucosal eosinophils displayed higher degranulation profile in IBS-D as compared to HC, with increased content of CRF in the cytoplasmic granules, which significantly correlated with IBS clinical severity, life stress background and depression. Eosinophils responded to substance P and carbachol by increasing secretory activity and CRF synthesis and release, without promoting pro-inflammatory activity, a profile similar to that found in mucosal eosinophils from IBS-D. Collectively, our results suggest that intestinal mucosal eosinophils are potential contributors to stress-mediated gut dysfunction through CRF production and release.

## Introduction

Eosinophils are multifunctional resident granulocytes of the gastrointestinal (GI) mucosa. These cells play a prominent role in the physiology and host responses contributing to mucosal homeostasis and inflammation trough the release of a wide plethora of mediators including cationic proteins, cytokines, chemokines, growth factors and lipids^1^. Eosinophils play a key role in allergic disease, and in responses to parasite and bacterial infections^2^. In the GI tract, the presence of elevated eosinophil counts is a feature of eosinophil GI disorders^2^, inflammatory bowel disease (IBD)^3^, and severe functional dyspepsia^4^. Eosinophil contribution to GI pathology seems to be mediated mostly by inflammatory mechanisms and by classical and non-classical food allergy-mediated activation^5^. However, eosinophils also synthesize neuro-hormones and peptides and express receptors that may contribute to both, regulatory physiology and disease mechanisms. In fact, intestinal mucosal eosinophils contain substance P (SP), vasoactive intestinal peptide (VIP), calcitonin gene-related peptide and corticotropin-releasing factor (CRF)^1,6,7^ and have recently been identified as potential contributors to the opioid and cannabinoid systems by playing a compensatory role^8^. Moreover, as most immune cells in the *lamina propria*, eosinophils locate near nerves in pathological conditions^9^, which facilitates bidirectional communication between the immune and nervous systems. Although, similar nerve-immune cell interactions, like the mast cell-nerve axis, have been shown to be relevant in GI dysfunction^10^, particularly in irritable bowel syndrome (IBS), the contribution of eosinophil-mediated neuro-immune mechanisms to GI dysfunction is not well understood.

Compelling evidence has accumulated pointing to a prominent role of stress in the pathophysiology of IBS and have identified stress and anxiety as important comorbid and permissive factors in the development and/or exacerbation of IBS symptoms^11,12^. Central CRF is the nuclear mediator of the neuroendocrine, immunological, autonomic, behavioral, and visceral responses to stress. However, peripheral CRF signaling also contributes to the GI stress response, regulating intestinal permeability, ion secretion, motility, inflammation and pain perception^13^, partly through mast cell recruitment and activation^14^.

Another noteworthy fact in the study of the origin of symptoms in IBS has been the transition from the old view of IBS as a motor disorder of the colon afflicting mostly stressed or anxious people, to the current view of IBS that contemplates a quite complex and multifactorial disorder, in which the participation of the small intestine acquires much more relevance^15,16^. Functional and pathologic studies by our group and others demonstrate the presence of low-grade mucosal inflammation, immune activation and intestinal epithelial disturbances in both upper and lower distal small bowel in IBS, in association with psychological stress, particularly in diarrhea-predominant IBS (IBS-D) patients^17–19^. In this scenario, eosinophils may act as mediators of local responses, by promoting immune activation, neural stimulation and barrier regulation. However, the role of eosinophils and the mechanisms underlying the molecular basis of these abnormalities are still unclear. We hypothesized that jejunal mucosal eosinophils contribute to IBS-D pathophysiology through CRF production and, therefore, aimed to identify jejunal eosinophil activity and contribution to IBS-D pathophysiology.

## Methods

### Participants and clinical assessment

Newly diagnosed IBS-D patients fulfilling the Rome III criteria^20^ were prospectively recruited from the outpatient gastroenterology clinic. Healthy controls (HC) were recruited from the general population by public advertising. Prior to entering the study, participants were asked to complete structured clinical questionnaires to verify the lack of symptoms in HC and to characterize digestive symptoms and to exclude other GI diseases. Dyspepsia was also evaluated following the Rome III criteria^20^. Past episodes of infectious gastroenteritis and GI comorbidities were assessed by means of broad biochemical and serological tests including anti-transglutaminase antibodies and thyroid hormones. An allergy specialist evaluated participants to discard food and respiratory allergy by clinical history and by a battery of skin prick tests (SPT) (Laboratorios Leti SA, Barcelona, Spain) for 22 common food allergens and 12 inhalants, as previously detailed^19^. Inclusion and exclusion criteria are summarized in Supplementary Table S1.

Clinical questionnaires were completed daily (10 days prior to biopsy collection) by all participants and recorded: (1) Severity of abdominal pain, according to a numeric rating scale (NRS), from 0 (no perception) to 10 (severe pain)^21^; (2) pain frequency (number of days with pain)^22^; (3) stool frequency (maximum number of bowel movements); (4) stool form (by the Bristol Stool Chart score)^23^; and (5) distention using a 6 point visual analog scale (VAS) ^22^. Background stress and depression scores were assessed using the validated Spanish versions of the Modified Social Readjustment Scale of Holmes-Rahe^24^, the Perceived Stress Scale of Cohen^25^, and the Beck’s Depression Inventory^26^, respectively. The severity of IBS was evaluated by the IBS severity scoring system (Francis Score)^22^. Written informed consent was obtained from each participant. The protocol was approved by the Ethics Committee at Hospital Vall d´Hebron [PR(AG)76/2006] and the study was carried out in accordance with the guidelines of the Declaration of Helsinki and the principles of good clinical practice.

### Experimental design and procedures

Clinical assessment and a jejunal biopsy with Watson's capsule were obtained in all participants as previously described^17^. Biopsy was immediately split into two similar pieces. One fragment was fixed in 4% buffered formalin and embedded in paraffin for further microscopic examination. The remaining fragment was stored at – 80 °C until processed for RNA isolation and gene expression analysis (microarray and qRT-PCR for eosinophil-associated genes) or processed for eosinophil ultrastructure assessment and CRF immuno-gold labeling by transmission electron microscopy (TEM). Samples for each experimental procedure were coded and analyzed blindly. Mechanistic analysis of CRF expression and release by SP or carbachol (CCh) were assessed in the human 15HL-60 differentiated to eosinophils cell line.

### Analytical procedures

#### RNA isolation and microarray analysis

Jejunal mRNA was isolated using the RNAeasy Mini Kit (Qiagen), following the manufacturer’s instructions. DNase treatment (Qiagen) was performed on columns on all samples at room temperature. RNA was eluted in 30 μL of RNase-free water and quantity and quality were confirmed by capillary electrophoresis (Agilent 2100 Bioanalyzer; Agilent Technologies). Only samples with values of RIN ≥ 7 were used. Microarray technology (Affymetrix human gene 1.1 ST) was performed on randomly-selected mucosal biopsies as previously described^17^. The complete data set is available at the NCBI Gene Expression Omnibus (https://www.ncbi.nlm.nih.gov/geo; accession number GSE14841). Differentially-expressed genes (*P* < 0.05), with a mean fold change of ≤ 0.7 and ≥ 1.4, were submitted to pathway and network analysis using the Ingenuity Pathway Analysis methodology (IPA 7.0, Ingenuity Systems) as previously described ^17^.

#### c-DNA synthesis and quantitative real time PCR

cDNA synthesis was performed using 1 μg of total RNA with the High Capacity Reverse Transcription Reagents Kit (Applied Biosystems, Thermo-Fisher Scientific) following manufacturer’s protocol and gene expression was assessed for a selection of genes using TaqMan assay. Gene expression was assessed for a selection of genes involved in eosinophil chemotaxis (CCL11, CCL24, CCL26 and CCR3), eosinophilic cationic proteins (PRG2, RNASE2, RNASE 3, EPX and CLC), survival factors (GM-CSF and IL5) and genes involved in secretory activity (SNAP23, VAMP2 and STX4), using validated TaqMan Gene Expression Assays (GEA) and the human 18S subunit ribosomal RNA gene as the endogenous control (Applied Biosystems). Quantitative real-time PCR (qPCR) was performed on an ABI PRISM 7500 FAST Sequence Detection System (Applied Biosystems). Due to the low abundance of eosinophil-specific genes in mucosal samples, pre-amplification of cDNA was performed before qPCR in the genes involved in chemotaxis (except CCL11) and eosinophil cationic proteins with the PreAmp Master Mix following the manufacturer’s instructions (Applied Biosystems). Each sample, including distilled water as negative control, was run in triplicate and data were analyzed by the 2^−ΔΔCt^ method. The expression of each gene was normalized to the endogenous control with constant expression and the fold-change was calculated individually with respect to the average of the HC group^18^.

#### Immunohistochemistry

Tissue Sects. (4 μm) were processed following standard procedures and stained with anti-human MBP or CD3 for eosinophils or intestinal epithelial lymphocytes (IELs) identification, respectively, following incubation with secondary antibody (table S3) and revealed by Vectastain ABC kit (Vector Laboratories, United Kingdom). Positive cells were quantified in 8–10 non-overlapping fields and results are expressed as the number of eosinophils per mm^2^ and lymphocytes per 100 epithelial cells, using the CellSens-1.7 software (Olympus BX61).

#### Transmission electron microscopy

##### *Ultrastructure of mucosal eosinophils*

Biopsies were fixed dehydrated, and embedded in Epon resin following standard protocols to evaluate the ultrastructure under transmission electron microscopy as previously described^18^. Ultrathin sections (80 nm) were examined using a Hitachi H-1,400 microscope at 75 kV equipped with a MegaView III camera (Soft Imaging System). A morphometric analysis was performed in all eosinophils identified in at least 24 sections per sample at 10,000–15,000× magnification. Eosinophil activation was assessed by quantifying the type and degree of degranulation (loss of granular content) and expressed as the percentage of degranulated area with respect to the total granular area. Eosinophils were categorized into: resting (containing no degranulated granules), low (containing 0– < 20% of granules degranulated) moderate (20–60%) and high (> 60%) degree of degranulation, as previously described^27^. The number of cytoplasmic granules and lipid bodies was also quantified. Measurements were performed using the ImageJ software (National Institutes of Health).

#### Evaluation of CRF in the intestinal mucosa by immuno-gold

CRF labelling was performed with the post-embedding immuno-localization method in Lowicryl HM20 resin sections. Ultrathin sections placed in carbon coated gold grids were blocked with 1% BSA/PBS, incubated with a mouse antihuman CRF antibody overnight at 4ºC, followed by a secondary anti-mouse antibody coupled to 15 nm gold nanoparticles (table S3). Quantification of CRF labelling was made in all mucosal eosinophils from at least 16 sections per sample at 80,000–100,000× magnification and is expressed as number of gold nanoparticles per granule. Prior to quantification of CRF in intestinal samples, the specificity of the primary antibody was validated by a dot blot assay on rat brain extract and the human eosinophil cell line (AML 14.3D1). Immunostaining protocol optimization was assayed in human jejunal samples and the eosinophil cell line. Experimental procedures and validation analysis are detailed in the supplementary material (Figures S1–S4).

#### Eosinophil response to neuropeptides

The human myeloid leukemia 15HL-60 (ATCC, CRL-1964) cell line was maintained and stimulated with the neuropeptides SP or CCh and with lipopolysaccharide (LPS) as control for eosinophil activation, as described in supplementary data. Concentration of stimulators was selected based on the literature^6,7,28^. After neuropeptide exposure, cells were collected at different time-points and examined for: 1) eosinophilic proteins gene expression, by quantitative PCR analysis as previously described, and data analyzed using basal condition as reference sample; 2) secretory activity of SNAP23 and VAMP2 by immunofluorescence following standard procedures (supplementary material); and 3) CRF gene expression, protein location and content, by qPCR, immunofluorescence and/or flow cytometry analysis, respectively. Experimental protocols are detailed in the supplementary material.

### Statistical analysis

Parametric data are expressed as mean ± standard deviation and comparisons between groups were performed by the unpaired Student’s t test (two-tailed). Non-parametric data are given as median (range). Comparisons between groups were done by the Mann–Whitney *U* test. Categorical variables were compared using the Fisher’s exact test or the Chi-square test. Relationships between clinical variables and biological variables were assessed by Spearman’s rank correlation analysis. *P* values ≤ 0.05 were considered significant and were adjusted for multiple comparisons using the Benjamini and Hochberg method^29^ and the application of correction is indicated in each table. In cell lines, non-parametric distribution was assessed by Kruskal–Wallis test followed by Dunn’s multiple comparison post-hoc test. Statistical analysis of time-course experiments was performed by two-way ANOVA followed by Bonferroni multiple comparison pot-hoc test.

## Results

### Study population

Twenty-five HC and 42 IBS-D patients were included in the study. There were no differences in age between participants; however, the number of women in IBS-D was higher than in HC (*P* < 0.05). Similar to our previous studies^17,18^, the HC group displayed comparable stress level in the last year as compared with the IBS-D group, which exhibited higher level of stress in the last month (*P* < 0.0001) and of depression (*P* < 0.0001) than HC subjects (Table 1). In the IBS-D group, 53% had dyspepsia, with similar frequency and intensity of abdominal pain, number of bowel movements and stool form than non-dyspeptic patients with IBS-D.

**Table 1:** Clinical and demographic characteristics of participants.

	HC (n = 25)	IBS-D (n = 43)	*P* value
Gender, F:M	11:14	30:13	0.036*
Age, years	35 (30–39)	37 (34–41)	0.242
Atopy, yes: no	6:14	20:18	0.253
Intensity of abdominal pain, score	0	47.69 ± 24.54	–
Frequency of abdominal pain, score	0	5.48 (3–10)	–
Bowel movements (n)	1 (1–2)	4 (1–12)	< 0.0001**
Stool form, Bristol score	3.3 (2–5)	5.5 (2–7)	< 0.0001**
Dyspepsia	–	23/43	
Distention	–	47.5 (0–100)	–
Holmes-Rahe Scale	116 (29–267)	179 (25–889)	0.237
Cohen Scale	16 (4–28)	25 (9–46)	< 0.0001**
Beck’s Depression Index	3 (0–22)	11 (0–36)	< 0.0001**

Values represent the median (range) or the mean ± SD. *P* Values considered significant are shown in bold. *P* value adjusted (false discovery rate): *P < 0.05; **P < 0.001. F, female; M, male; HC, healthy controls; IBS-D, diarrhea-predominant IBS.

### Distinctive transcriptional profile linked to eosinophil signaling and activation in IBS-D

Consistent with our previous work^17,30^, the analysis of the differential mucosal transcriptome revealed canonical signaling pathways key for intestinal barrier function and immunological functions as significant in IBS-D^17^. Interestingly, biological functions related to eosinophil chemotaxis, survival and activation appeared as differentially expressed between the two groups (*P* < 0.05) (figure S5).

### Decreased pro-inflammatory activation profile of mucosal eosinophils in IBS-D

**Figure 1:**
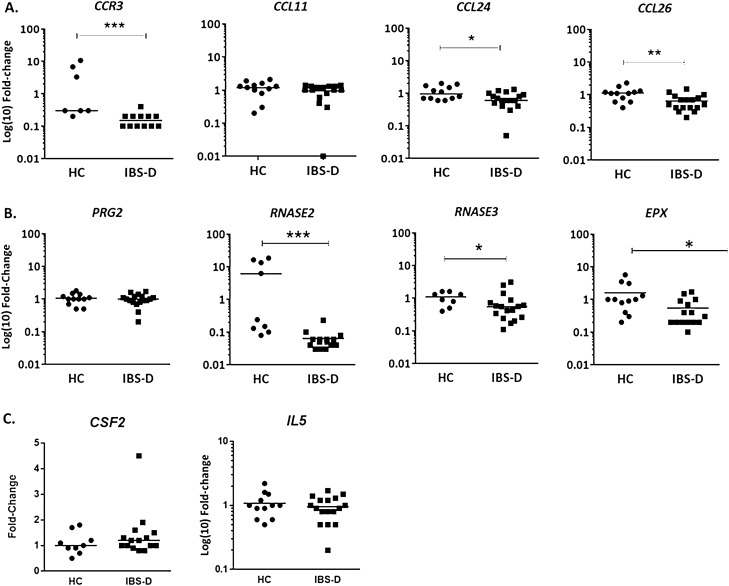
Expression of genes associated with eosinophil recruitment, proinflammatory and secretory activity. Gene expression analysis of (**A**) chemotactic (**B**) cationic and (**C**) survival eosinophil-related genes. Individual values represent the fold change with respect to the average of the HC group. C–C chemokine receptor 3 (*CCR3*), C–C motif chemokine 11, 24 and 26 (*CL11*, *CCL24* and *CCL26,* eotaxins 1, 2 and 3, respectively), colony stimulating factor 2 (CSF2); eosinophil major basic protein (MBP, Proteoglycan 2, *PRG2)*, Charcot-Leyden crystal galectin *(CLC),* eosinophil-derived neurotoxin (ribonuclease A family member 2, *RNASE2*), eosinophil cationic protein (ribonuclease A family member 3, *RNASE3*), eosinophil peroxidase (EPX); interleukin-5 (IL-5). *P* value adjusted (false discovery rate): *P < 0.05, **P < 0.01; ***P < 0.001. HC, healthy control; IBS-D, diarrhea-predominant IBS (HC, n = 7–12; IBS-D, n = 12–17).

To examine tissue eosinophil infiltration and location, eosinophil counts were analyzed only in specimens with well-preserved villous architecture. Routine histopathological analysis revealed the absence of viral inclusions or parasitic infection and a discrete lymphoplasmacytic infiltrate in the *lamina propria.* The number of intraepithelial CD3^+^ cells [HC:25.2 ± 10.2; IBS-D:26.3 ± 18.5 lymphocytes/100 epithelial cells] and *lamina propria* eosinophils [HC: 44 ± 7; IBS-D: 83 ± 19 cells/mm^2^] was similar in both groups. For validation of microarray data and IPA analysis, genes involved in eosinophil chemotaxis, activation, survival and secretory activity were further quantified by qPCR. Regarding eosinophil homing to the gut, eotaxin receptor *CCR3* and eotaxins 2 and 3 (*CCL24* and *CCL26*, respectively), were down-regulated in IBS-D patients, with no differences in eotaxin 1 (*CCL11*) (Fig. 1A). Gene expression of eosinophil pro-inflammatory cationic proteins: eosinophil-derived neurotoxin (EDN (*RNASE2)*), eosinophil cationic protein (ECP (*RNASE3)*) and eosinophil peroxidase (EPO (*EPX)*), were also down-regulated in the intestinal mucosa of IBS-D patients, while no changes in MBP (*PRG2)* was observed compared to HC (Fig. 1B). The expression of GM-CSF and IL-5 (genes related to eosinophil survival) were similar in both groups (Fig. 1C).

### Increased secretory profile of mucosal eosinophils in IBS-D

**Figure 2:**
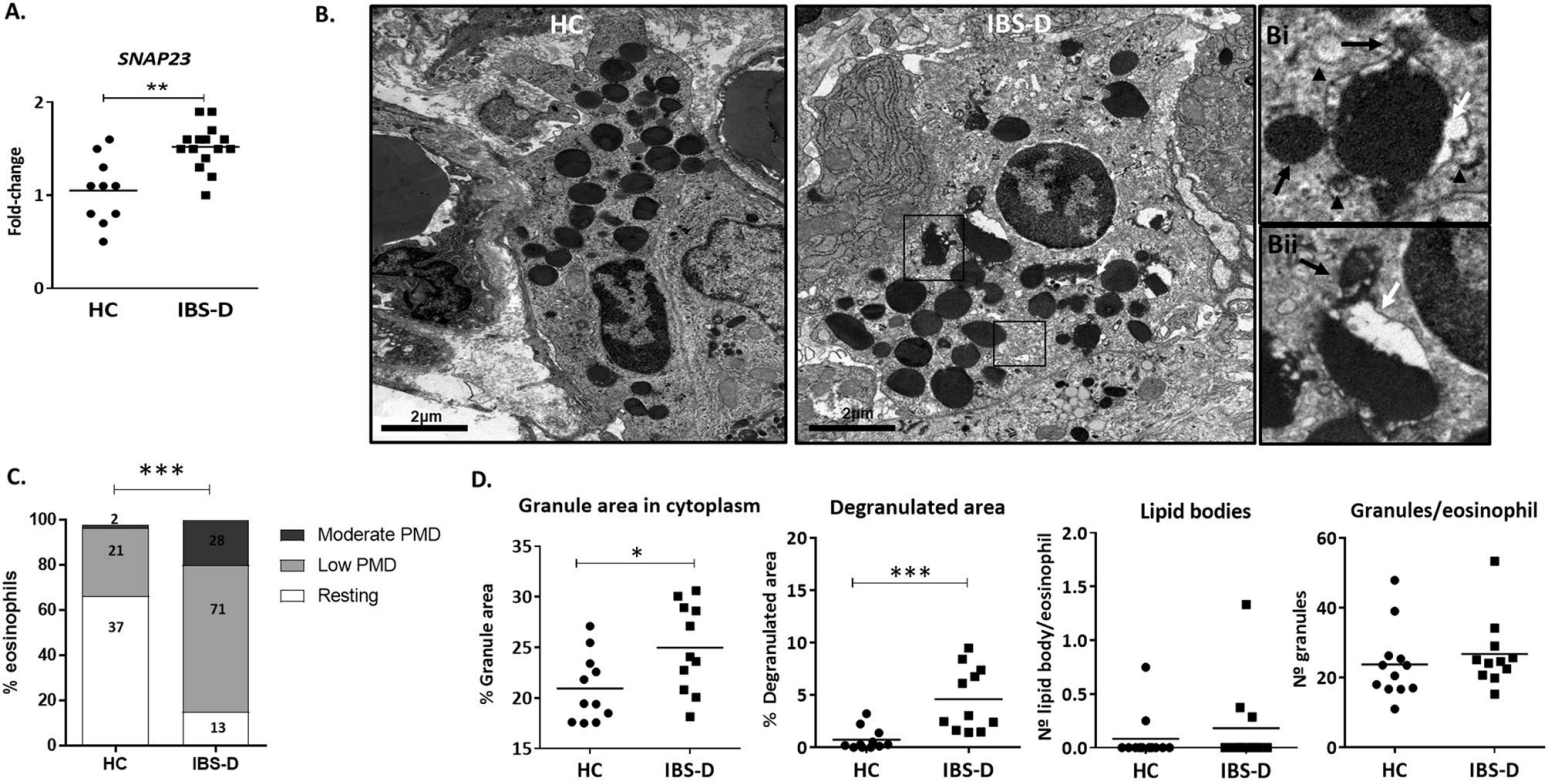
Assessment of mucosal eosinophil activation. (**A**) Quantitative gene expression analysis of the secretory activity-related synaptosomal-associated protein 23 (*SNAP23*). Individual values represent the fold change with respect to the average of the HC group (HC, n = 10; IBS-D, n = 16). (**B**) Representative images of mucosal eosinophils. White arrow indicates loss of granular content of cytoplasmic granule. The boxed areas in (**B**) are shown in (Bi-Bii) at higher magnification. Black arrowhead in the insert Bi (in IBS-D) shows a partially degranulated granule with vesico-tubular structures and sombrero-like vesicles indicative of active degranulation. Black arrow in Bi and Bii indicates granule enlargement, also indicative of PMD process Magnification: bars indicate 2 µm. (**C**) Quantification of degree of PMD. Percentage of eosinophils with moderate, low or no degranulation (HC, n = 13; IBS-D, n = 11). (**D**) Percentage of granular cytoplasmic area and the degranulated area of granules in eosinophils in each group, and number of lipid bodies and granules per eosinophil. Individual values represent an average of counts in different eosinophils in different sections of each sample *P < 0.05; ***P < 0.001. HC, healthy control; IBS-D, diarrhea-predominant IBS (HC, n = 11–12; IBS-D, n = 11).

To further assess secretory activity in the jejunal mucosa, the expression of SNAREs genes involved in eosinophil secretion process were assessed. *SNAP23,* a membrane protein related to vesicular transport, was up-regulated in IBS-D, suggesting an increased secretory activity in this group (Fig. 2A), despite no differences were observed in other eosinophil-related SNARE proteins syntaxin 4 (*STX4,* HC: 1.01 ± 0.24; IBS-D: 0.98 ± 0.23 fold change*)* and vesicular-associated membrane protein 2 (*VAMP2*, HC: 1.03 ± 0.21; IBS-D: 0.96 ± 0.24 fold change).

Qualitative analysis of the eosinophil ultrastructure revealed signs of activation and secretory activity in eosinophils from IBS-D. Some cytoplasmic granules were partially empty with non-fused secretory granules, indicative of piece-meal degranulation (PMD) with higher frequency and selective fragmentation of granule core (Fig. 2B), higher frequency of vesico-tubular structures such as “sombrero-like” vesicles (Fig. 2B Bi) and signs of granule enlargement and disarrangement of granule cores and matrices (Fig. 2B Bi and Bii). By the use of a quantitative PMD index previously defined as the percentage of granules with PMD^27^, eosinophils were classified as resting eosinophils, or displaying low/moderate/high PMD degranulation. From a comparable number of samples (HC n = 13; IBS n = 11, table S3), similar number of eosinophils [60 for HC; 73 for IBS-D] and cytoplasmic granules [1665 for in HC; 1785 for in IBS-D] were analyzed in each group. Quantitative analysis revealed 62% of resting and 38% of degranulated eosinophils in HC, of which 91% showed a low degree of PMD; while the IBS-D group showed 17% of resting and 83% of degranulated eosinophils, of which 72% eosinophils displayed low PMD and 28% moderate PMD (Fig. 2C; *P* < 0.0001). The analysis of morphological activation markers showed larger cytoplasmic area occupied by granules (i.e. cytoplasmic granular density) in the IBS-D group (*P* < 0.05), with also increased degranulated area (*P* < 0.0001), as compared to control samples (Fig. 2D). No differences were observed in the number of cytoplasmic granules and lipid bodies per eosinophil between groups (Fig. 2D).

### Differential granular CRF content in mucosal eosinophils and mucosal *CRHR1* expression in IBS-D

**Figure 3:**
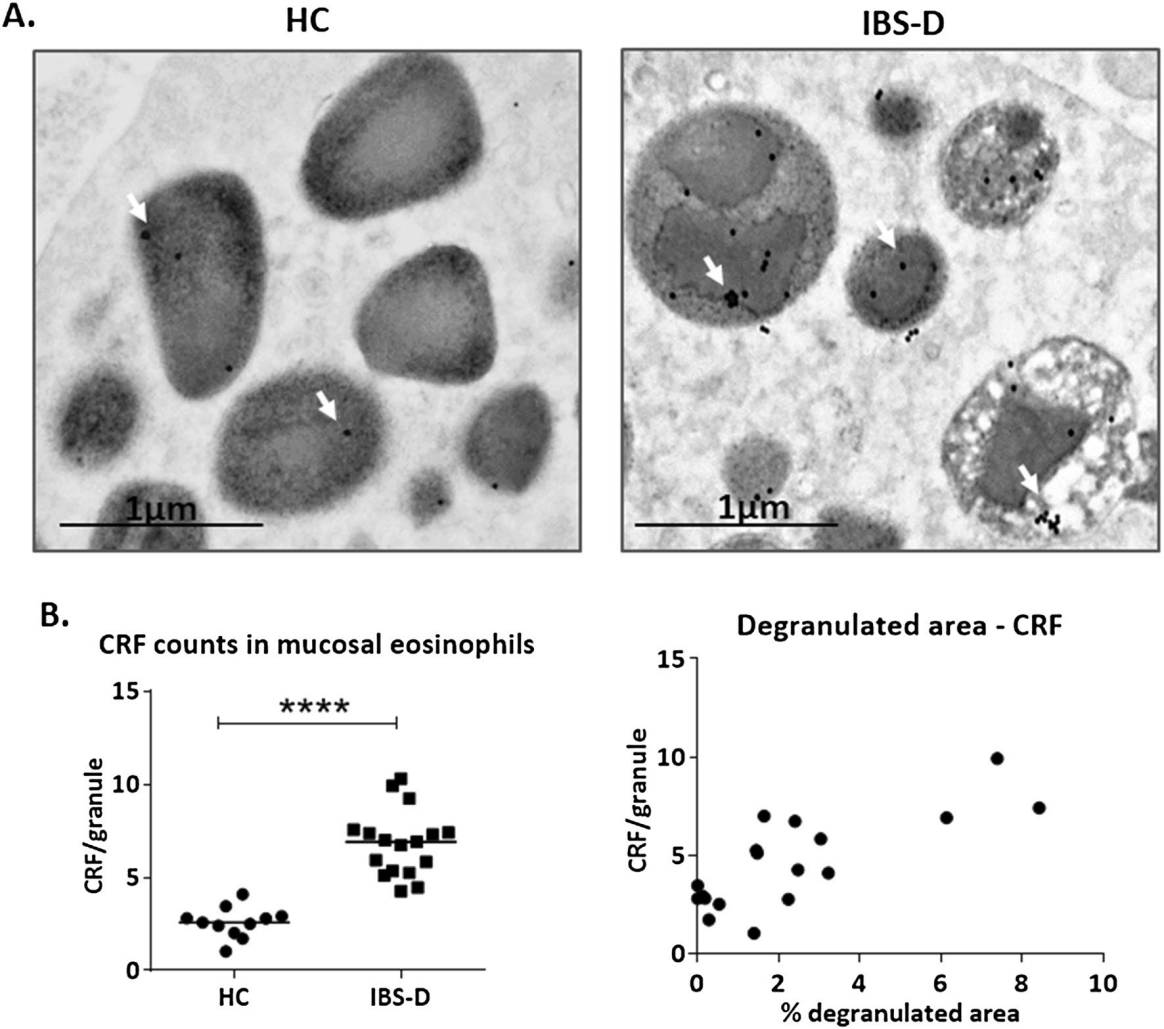
Localization and quantification of CRF immunolabelling and CRF receptor expression. (**A**) Representative micrographs of CRF positivity in eosinophils. (**B**) Left: CRF content in eosinophil granules. Particles indicate the detection of human CRF by means of nanogold particles (see “Material and methods”). Right: Correlation between CRF content and degranulated area of eosinophils. The r_s_ and *P* values according to Spearman rank correlation are indicated. Individual values represent an average of CRF counts of different sections of each sample ****P < 0.0001. HC: healthy control; IBS-D: diarrhea-predominant irritable bowel syndrome (HC, n = 11; IBS-D, n = 17).

An exhaustive ultrastructural analysis of all *lamina propria* resident cells of the intestinal mucosa revealed that positivity to CRF was identified only in eosinophils (figure S3). CRF signal was identified as gold-electron-dense particles and was found in cytoplasmic granules of eosinophils in both, IBS-D patients and HC (Fig. 3A). Comparable numbers of eosinophils per sample and number of granules per eosinophil were analyzed in both groups (figure S6). Interestingly, signal quantification revealed a significantly higher CRF content per granule in eosinophils from IBS-D samples (Fig. 3B left), which positively correlated with the percentage of degranulated area of eosinophils (Fig. 3B right). Additionally, the gene expression of CRF receptors (*CRHR*) in the jejunal mucosa was analyzed in both experimental groups. *CRHR1* expression was down-regulated in IBS-D patients (IBS-D: 0.587 ± 0.431; HC: 1.08 ± 0.340 fold change; *P* < 0.01), while no differences in *CRHR2* were detected between groups.

### Association between mucosal eosinophil CRF content and *CRHR1* expression with IBS-D clinical manifestations

To gain insight into eosinophil-derived CRF connection to intestinal dysfunction, we then analyzed the association between CRF granular content in mucosal eosinophils, *CRHR1* gene expression and major clinical manifestations. A significant positive correlation was found between CRF content and IBS-D symptoms including abdominal pain intensity, IBS severity, distention, number of bowel movements and stool form. Notably, psychological stress and depression symptoms also positively correlated with CRF content (Table 2).

**Table 2:** Correlation between CRF content in eosinophils and clinical parameters.

CRF/granule	r_s_	*P* value	FDR	HC	IBS-D
Intensity of pain	**0.799**	**0.0002**	0.001***	–	17
Frequency of pain	0.3812	0.1313	0.184	–	17
IBS Severity	**0.5343**	**0.0291**	0.058*	–	17
Distention	**0.4856**	**0.0499**	0.087*	–	17
Dyspepsia	0.3114	0.2454	0.312	–	17
Bowel movements	**0.6161**	**0.0013**	0.005**	7	17
Stool form	**0.6055**	**0.0017**	**0.005**	7	17
Stress (last month)	**0.6557**	**0.0001**	0.001***	12	17
Stress (last year)	0.3478	0.0645	0.100	12	17
Depression score	**0.7191**	** < 0,0001**	0.001***	12	17
Eosinophil /mm^2^	0.1404	0.4849	0.522	11	16
Atopy	− 0.06747	0.7433	0.743	10	16
Sex	**0.4435**	**0.016**	0.037*	12	17

*P* values considered significant are shown in bold. *P* value adjusted (false discovery rate (FDR)): *P < 0.05; **P < 0.01 and ***P < 0.001. HC, healthy controls; IBS-D, diarrhea-predominant IBS; A FDR < 10% was accepted as significant.

The correlation observed between CRF and sex is affected by the higher female prevalence in the IBS group, but differences between groups are not driven by a sex effect, as both, men and women have significantly higher amount of CRF in eosinophil granules in IBS-D respect to HC (figure S7). Moreover, *CRHR1* expression was significantly associated with clinical manifestations showing negative correlation with intensity of pain and stool form, as well as with psychological stress and depression symptoms (Table 3).

**Table 3:** Correlation between CRFR1 gene expression in jejunal samples from HC and IBS-D and clinical parameters. *P* values considered significant are shown in bold. *P* value adjusted (false discovery rate (FDR)): *P < 0.05; **P < 0.01. HC, healthy controls; IBS-D, diarrhea-predominant IBS; A FDR < 10% was accepted as significant.

*CRHR1* expression	r_s_	*P* value	FDR	HC	IBS-D
Intensity of pain	−** 0.6157**	**0.0109**	0.0681*	–	17
Stool form	−** 0.5743**	**0.0042**	0.0357**	7	17
Stress (last year)	−** 0.5561**	**0.0026**	0.0357**	12	16
Depression score	−** 0.4646**	**0.0143**	0.0620*	11	16
Stress (last month)	− 0.3552	0.075	0.2108	11	16

### Neuropeptides mediate CRF production and release without induction of a proinflammatory profile in eosinophils

**Figure 4:**
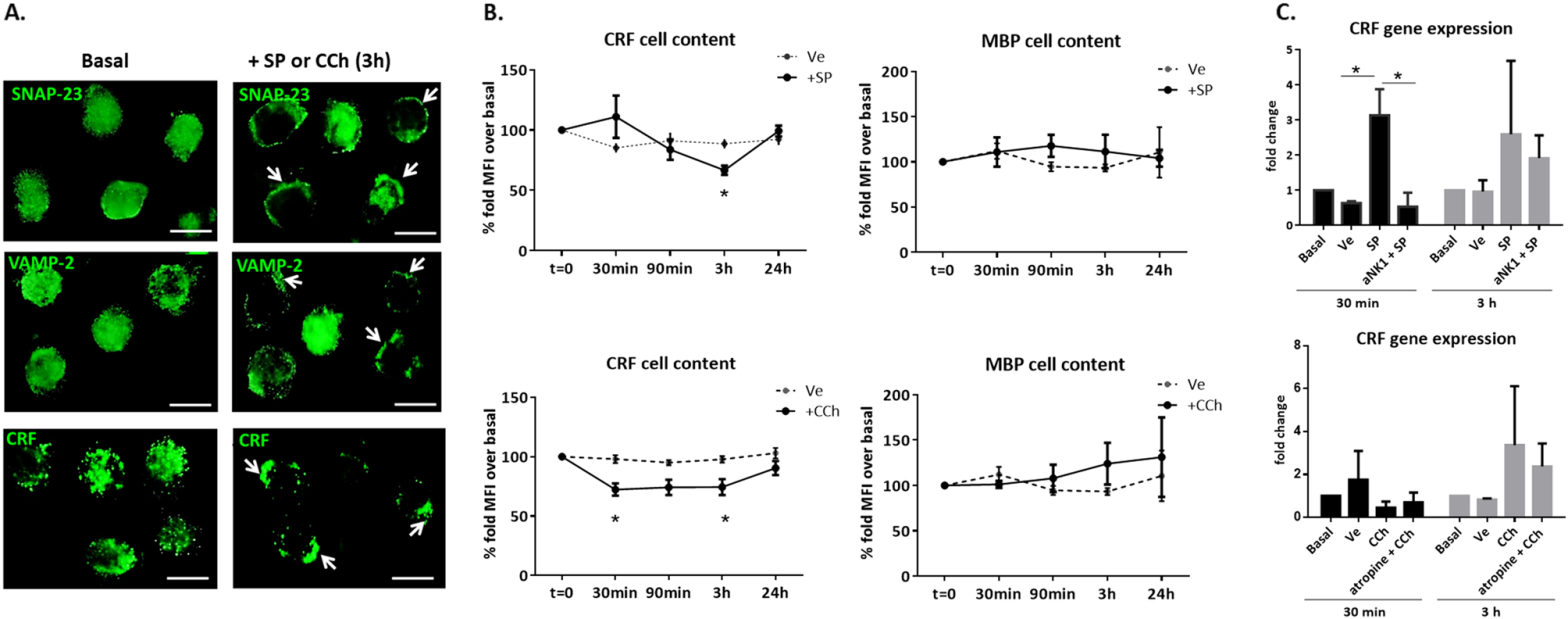
Localization of CRF in the eosinophil 15HL-60 cell line after exposure to neuropeptides. (**A**) Representative images of SNAP-23, VAMP-2 and CRF localization at basal and after exposure for 3 h to SP or CCh (one image is shown, as exposure to both neuropeptides yielded similar results). Fluorescence signal in the cytoplasm at basal conditions and relocated (white arrows) to the plasma membrane after exposure to SP or CCh. Images representative of 3 independent experiments, run in triplicate. Bars indicate 10 μm. (**B**) Intracelluar CRF and MBP content over the time course exposure to SP or CCh identified by flow cytometry. Percentage of fold mean fluorescence intensity (MFI) change over basal condition expressed as mean ± standard error (n = 8 independent experiments for SP and CCh). (**C**) CRF gene expression at 30 min and 3 h after exposure to neuropeptides. Fold-change calculated by normalizing data with the endogenous control gene (*PPIA*), comparing each time point to untreated cells. Graphs represent results from 3 independent experiments, run in triplicate. Data are expressed as median (range). *P < 0.05; SNAP23: Synaptosomal-associated protein 23; VAMP-2: Vesicle-associated membrane protein 2; SP: Substance P; CCh: carbachol; CRF: corticotropin releasing factor; Ve: vehicle.

In order to identify neuro-modulation of eosinophil activity, mature eosinophils (15HL-60 cell line) were exposed to SP or CCh and SNAP23 and VAMP2 redistribution in cells was analyzed, together with CRF production and release. After 3 h of SP or CCh exposure, SNAP23 and VAMP2 migrated from cytoplasm to the plasma membrane, indicating active degranulation (Fig. 4A). A quantitative analysis of CRF content by flow cytometry revealed a significant decrease of intracellular CRF, 30 min after exposure to CCh and at 3 h of exposure to SP (Fig. 4B). Degranulation was confirmed by redistribution of granules towards the membrane together with a decrease in the fluorescence intensity of CRF signal (Fig. 4A).

To assess the effect of neuropeptide stimulation on inflammatory mediators, analysis of MBP content revealed no changes after stimulation with SP or with CCh (Fig. 4B), suggesting neuropeptides specifically modulate CRF release.

To further evaluate the effect of neuropeptides on eosinophil CRF production and its specificity, CRF gene expression was quantified in parallel to antagonist pre-treatment. CRF mRNA expression was up-regulated after 30 min of SP exposure and rescued in cells pre-treated with antagonist, while no statistical differences after CCh treatment were observed (Fig. 4C). Notably, nor SP neither CCh upregulated gene expression of eosinophil cationic proteins, unlike LPS exposure, used as positive control of inflammatory pathways activation (figure S8).

## Discussion

This novel study demonstrates increased content of CRF in jejunal eosinophils in IBS-D, as compared to health, content that positively correlates with severity of major clinical parameters of GI dysfunction and the degree of psychological stress and depression. Interestingly, IBS-D and HC display similar number of eosinophils infiltrating the jejunal mucosa but eosinophils in IBS-D show higher CRF granular content and degranulation features than HC. These findings, together with previous studies, suggest that mucosal eosinophils play a role in the origin of symptoms in IBS-D, possibly via peripheral stress-mediated intestinal dysfunction^7,31^.

The analysis of integrative pathways associated with the transcriptome of IBS-D identified eosinophil chemotaxis, activation and survival, as significant pathways differentially expressed compared to HC. This is consistent with the participation of eosinophils in the modulation of intestinal inflammation, as has been shown in several GI eosinophilic disorders and IBD^32^, in which mucosal eosinophils have been identified as pro-inflammatory cells due to their content in pleiotropic cytokines, chemokines and toxic cytoplasmic proteins^33^. Contrary to what has been reported in previous studies in the intestinal mucosa of IBS, in which a discrete increase in numbers of other immune cells such as mast cells, plasma cells and lymphocytes is rather frequent^18,19,34,35^, in the present study, however, we observed equal eosinophils counts between groups. In post-infectious-IBS, however, mucosal eosinophil counts are significantly increased in the cecum^36^, while in constipation-predominant IBS reduced number of mucosal colonic eosinophils have been shown^37^, suggesting different pathogenic mechanisms in IBS subtypes, findings that deserve further investigation. All those results reinforce the idea of the relative importance of counting cells as compared to measuring activity. In this sense, we have observed down-regulation of chemotaxis genes and *CCR3* gene expression in IBS-D, together with a decrease in *EPX* (EPO), *RNASE2* (EDN) and *RNASE3* (ECP) eosinophilic proteins expression, without changes in eosinophil survival factors (GM-CSF and IL-5). Unfortunately, the limited amount of sample did not allow us to quantify eosinophilic proteins to further validate gene expression analysis and confirm the activation profile of mucosal eosinophils in IBS-D. While these analyses will be performed in future studies, a non-proinflammatory profile of eosinophils is supported by our in vitro results showing CRF synthesis and release by eosinophils exposed to neuropeptides and not MBP, a pro-inflammatory marker, together with EDN and ECP^38^. Altogether, our results suggest specific conditions for eosinophil recruitment and activation which may account for physiological eosinophil counts in IBS-D. Whether these findings are the result of existing of homeostatic loops, in part via eotaxin-CCR3 pathway, that prevent eosinophil-mediated inflammatory activity in the mucosa of IBS needs to be explored.

Eosinophils can modulate tissue activity through the extracellular release of a variety of granule-derived products. The up-regulation of SNAP-23 gene expression in IBS-D suggests an increase in eosinophil secretory activity involving the release of small and regulated amount of mediators^39^ a mechanism named PMD that is also present in mast cells and neutrophils^40^. Eosinophil PMD has been described in inflammatory conditions such as ulcerative colitis, allergic rhinitis and asthma patients^27^, by ultrastructural analysis as the loss of granular density without fusion of intergranular membranes and the increase in cytoplasmic secretory vesicles^41^. In those patients, the moderate to extensive degree of PMD^27^ and the presence of extracellular and fecal eosinophil-derived cationic proteins suggest an inflammatory activation profile of eosinophils^5,42–44^. In the present study, quantitative analysis demonstrated a low degree of PMD in jejunal eosinophils, being significantly higher in IBS-D respect to HC. The number and the area of cytoplasmic granules have also been described as relevant in eosinophil activation^45^. Hypodense eosinophils (that contain less or smaller granules) are particularly important in the pathophysiology of various inflammatory diseases such as hypereosinophilic syndrome^46^ or allergic rhinitis^47^, and are identified as activated due to enhanced eosinophil cytotoxic activity and degranulation^46,48^. Here, we show that eosinophils in IBS-D have similar number of granules as in health, but with increased granule area, not fulfilling, therefore, hypodense characteristics. Despite this observation needs to be further explored, it also supports a non-inflammatory activation profile of eosinophils in IBS-D.

Emerging research points to the eosinophil as a key factor in stress-mediated intestinal dysfunction because they can respond, store and release a wide variety of neuropeptides including CRF^1,7,49^. IBS is defined as a disorder of the brain-gut axis that is frequently associated with exaggerated response to stress events. About 50% of IBS patients display psychiatric comorbidities such as anxiety and depression and suffer more chronic stress than the healthy population^50^. The CRF system is the predominant mediator of the stress response, both in the brain and in the gut^51^. Evidence from clinical and experimental studies indicate that psychological stressors have a marked impact on intestinal sensitivity, motility, secretion and permeability^14,51^. Similar to preclinical studies of chronic psychological stress^52^, recent observations show that peripheral administration of CRF enhanced visceral sensorimotor function, epithelial permeability, hyperalgesia, mast cell activation and serotonin responses in health and in IBS patients^52^, acting through specific CRF receptors present in both, the small and the large intestine, and further supporting the contribution of local gut CRF signaling to the systemic response to stress. Although CRF has been detected at both the mRNA and protein levels in stimulated monocyte-derived dendritic cells^53^, enteric cells^54^ and colonic mucosal eosinophils^7^, its source in the small intestine is poorly studied. Here, we show for the first time that CRF in the jejunal mucosa is found only in eosinophil granules, in higher amount in IBS-D patients as compared to controls. Moreover, the amount of CRF per granule significantly correlates with IBS clinical manifestations, including abdominal pain and bowel habits, and with their severity, thereby suggesting a role for local CRF production by eosinophils in IBS origin. Our results are consistent with earlier findings in mice submitted to chronic restraint stress, in which the increase in CRF expression in eosinophils paralleled the stress exposure, decreasing gradually thereafter^6^, and also in human studies, where CRF levels were also associated with depression^55^. Neuromediators such as SP and acetylcholine and their receptors also play an important role in the modulation of stress responses in the central nervous system as well as in stress-induced intestinal inflammation^56,57^. Eosinophils express neuropeptide receptors and can interact directly with nerves in homeostasis and disease. In fact, eosinophil stimulation with SP or CCh induce eosinophils to release CRF^6,7^. The present study extends existing knowledge by revealing that neuromediators selectively induce synthesis and release of CRF by PMD in a non-pro-inflammatory pathway. Thus, the release of SP or acetylcholine from nerve endings may activate eosinophils to produce and release CRF in the intestine, mediating local stress responses in IBS-D^58,59^.

Although not tested here, a putative explanation for the association between CRF in eosinophils and gut dysfunction in IBS-D is mast cell activation. This study supports the hypothesis that peripheral CRF may act as the signaling system between eosinophils and mast cells in the pathogenesis of barrier dysfunction and symptom generation in IBS-D. However, additional experiments using inhibitors of CRF synthesis, specific antagonists of CRF receptors and mast cell stabilizers are needed to confirm this hypothesis. CRF receptors (CRF1 and CRF2) are widely distributed in the GI tract, and particularly present in mucosal mast cells^60^. CRF1 primes stress-mediated mast cell degranulation^61^ while CRF2 has been shown to act as a negative global modulator of mast cell degranulation and to reduce stress-induced increase in intestinal permeability in the mouse ileum^62^. In this study, we observed downregulation of *CRHR1* in IBS-D, which negatively correlated with pain and stool consistency and with chronic stress and depression. To our knowledge, CRF1 expression in the jejunal and colonic mucosa has been preliminarily reported in IBS^63^ and, more recently, shown to be down-regulated in IBS-D by us in a separate study^31^, though its specific function at this location has not been established yet. The finding that mast cell overstimulation with CRF leads to down-regulation of *CRHR1* in an in vitro model^64^ may explain why this receptor is decreased in IBS-D and negatively correlates with stress and depression levels, possibly because of chronic overstimulation. Interestingly, CRF1 has been demonstrated to mediate visceral hyperalgesia induced by repeated psychological stress in rats, and the use of a specific antagonist, antalarmin, or a non-selective CRF receptor antagonist, α-helical CRF, blocks stress-related changes in GI motility in animals^65,66^. However, despite promising preclinical results, the use of the specific CRF1 receptor antagonist, pexacerfont, in woman with IBS-D and anxiety did not significantly modify bowel function, anxiety or depression levels^67^. Notwithstanding, based on our findings, inhibition of eosinophil CRF production and/or release could be alternative therapeutic strategies to CRF receptors antagonism in IBS. Further studies are also needed to better define CRF receptors activity and its modulation by stress within the intestinal mucosa.

The importance of our findings needs to be related to the increasing recognition of the small bowel in the generation of symptoms in IBS, which can be ascribed to the distinctive anatomical structure and function of the small intestine, compared with the colon. The small bowel is equipped with a vast and versatile *lamina propria*-associated immune system; with a unique epithelial-associated hormone and neurotransmitter machinery; with a slow trending motor activity; with a particular morphological and ultrastructural composition of luminal tips and intercellular junctions that determine intestinal permeability, secretion, and absorption; with a differential gas and biliary metabolism; with a less deeper and fragile mucus layer; with a scarce but predominantly aerobic microbiota in its upper segments; and with an intricate pain processing system^68^. Therefore, in this context, our findings support and should stimulate future research focused on the role of neuro-immune interactions happening in the small bowel, as compared to the colon, in the pathogenesis of IBS.

In conclusion, our study reveals distinctive mucosal eosinophil activity in IBS-D, featured by higher CRF content respect to health, in association with GI severity, anxiety and depression. These data, together with previous observations, suggest a role for eosinophils in the pathophysiology of IBS-D via peripheral CRF. Targeting CRF in the small bowel intestinal mucosa may be a useful approach in the management of IBS patients, particularly those suffering high levels of stress, and of other functional GI disorders.

## Supplementary information

Supplementary information.

## Abbreviations

CCh: Carbachol
*CRHR*: Corticotropin releasing hormone receptor gene
CRF: Corticotropin releasing factor
ECP: Eosinophil cationic protein
EDN: Eosinophil-derived neurotoxin
GI: Gastrointestinal
HC: Healthy control
IBD: Inflammatory bowel disease
IBS: Irritable bowel syndrome
IBS-D: IBS with diarrhea
IBS-SSS: IBS severity scoring system
LPS: Lipopolysaccharide
MBP: Major basic protein
SP: Substance P
