# Supplementary material for: Overexpression of corticotropin-releasing factor in intestinal mucosal eosinophils is associated with clinical severity in Diarrhea-Predominant Irritable Bowel Syndrome
Source: Sci Rep. 2020 Nov 26;10:20706. doi: 10.1038/s41598-020-77176-x (PMC7692489; doi:10.1038/s41598-020-77176-x)
Supplement: Supplementary file 1 — Supplementary information. [file 41598_2020_77176_MOESM1_ESM.pdf]

Supplementary material

Overexpression of corticotropin-releasing factor in intestinal mucosal eosinophils is associated with clinical severity in diarrhea-predominant Irritable Bowel Syndrome

Authors:

Eloísa Salvo-Romero[1,2], Cristina Martínez[2], Beatriz Lobo[2], Bruno K. Rodiño-Janeiro[2], Marc Pigrau[2], Alejandro Sánchez-Chardi[3], Ana María González-Castro[1], Marina Fortea[1], Cristina Pardo-Camacho[1], Adoración Nieto[2], Elba Expósito[1], Danila Guagnozzi[1], Amanda Rodriguez-Urritia[4,5], Inés de Torres[6], Ricard Farre[7,8], Fernando Apiroz[2,8], Carmen Alonso-Cotoner[2,8], Javier Santos[2,8], María Vicario[1,8].

Laboratories of [1]Translational Mucosal Immunology and [2]Neuro-Immuno-Gastroenterology. Digestive System Research Unit, Vall d'Hebron Institut de Recerca, Department of Gastroenterology, [4]Department of Psychiatry, and Department of Pathology[6], Hospital Universitari Vall d'Hebrón, Universitat Autònoma de Barcelona, Barcelona, Spain. [3]Servei de Microscopia, Universitat Autònoma de Barcelona, Barcelona, Spain. [7]Translational Research Center for Gastrointestinal Disorders (TARGID), KU Leuven. Centro de Investigación Biomédica en Red de Salud Mental (CIBERSAM)[5] and Enfermedades Hepáticas y Digestivas (CIBEREHD)[8] Madrid, Spain.

A. Supplementary material and methods

Table S1. Inclusion and exclusion criteria.

Inclusion criteria	**HC**	**IBS-D**
Age: 18-60 years	yes	yes
Rome III criteria for IBS-D	no	yes
Naïve (newly-diagnosed)	no	yes
Acceptance of the study protocol	yes	yes
Exclusion criteria		
Clinical history of food allergy	yes	yes
Positivity to SPT to foodstuff	yes	yes
Infectious gastroenteritis	yes	yes
Gastrointestinal comorbidities	yes	yes
Pregnancy	yes	yes
Major psychiatric disorders	yes	yes
Use of medication (steroids, immunosuppressive drugs, anti-histaminic and mast cell stabilizers)	yes	yes

Table S2. Experimental procedures performed in intestinal samples from each experimental group

Participant	Experimental procedure	Participant	Experimental procedure	Participant	Experimental procedure
H1	1+2+3	IBS1	1+2+3	IBS26	2+3
H2	2	IBS2	2	IBS27	2+3
H3	1+2+3	IBS3	2+3	IBS28	2+3
H4	1+2+3	IBS4	1+2+3	IBS29	2+3
H5	2 + 3+ 4 + 5	IBS5	4 + 5	IBS30	1+2+3
H6	2+3	IBS6	4 + 5	IBS31	4 + 5
H7	2 + 3+4 + 5	IBS7	4	IBS32	4 + 5
H8	1+2 + 3+4 + 5	IBS8	4 + 5	IBS33	4 + 5
H9	4 + 5	IBS9	4	IBS34	4 + 5
H10	2+3	IBS10	4 + 5	IBS35	5
H11	2 + 3+ 4 + 5	IBS11	4	IBS36	4
H12	2 + 3+ 4 + 5	IBS12	4 + 5	IBS37	2
H13	2 + 4 + 5	IBS13	4	IBS38	2
H14	2+3	IBS14	4 + 5	IBS39	2
H15	4 + 5	IBS15	4	IBS40	2
H16	4 + 5	IBS16	4	IBS41	2
H17	4 + 5	IBS17	2+3	IBS42	2
H18	4 + 5	IBS18	1+2+3	IBS43	2
H19	4 + 5	IBS19	2+3		

H20	4 + 5	IBS20	1+2+3
H21	2	IBS21	2+3
H22	2	IBS22	2
H23	2	IBS24	2+3
H24	2	IBS23	2+3
H25	2	IBS25	2+3

1: gene expression by microarray; 2: gene expression by qPCR; 3: Eosinophil quantification by MBP Immunohistochemistry; 4: CRF quantification by Immuno-gold in TEM; 5: eosinophil granule area and degranulated area quantification by ImageJ on TEM images.

Table S3. Antibodies and dilution used

Antibody	***Supplier***	***Dilution***
Immunohistochemistry		
Monoclonal mouse anti-human CD3 (clone PS1)	Leica Novocastra	1/100
Polyclonal rabbit anti- human MBP	Abcam	1/50
Flow cytometry / Immunofluorescence		
Monoclonal goat anti-human CRF clone C-20	Sta Cruz Biotech.	1/50
Monoclonal mouse anti-human MBP clone BMK13	AbD Serotec	1/25
Polyclonal rabbit anti-human SNAP23	Abcam	1/50
Polyclonal rabbit anti-human VAMP2	Abcam	1/50
Electron Transmission Microscopy		
Monoclonal goat anti-human CRF clone C-20	Sta Cruz Biotech	
Secondary Ab rabbit anti-goat	British BioCell International.	

Validation of CRF immunolabeling:

Validation of the technique for CRF staining in human jejunal samples and eosinophilic cell line was confirmed before analysis, by assessing:

1) CRF antibody specificity

Goat anti-human CRF (table S3) was tested in dot blot assays using rat-brain tissue extract (Sigma) as positive control cell lysate, the AML14.3D10 cell line as negative

control and primary antibody absorbed as a control of reaction (figure S1). Dot blot was performed on nitrocellulose membranes with pre-absorbed lysates (5µL) and primary antibody (2µL) as a control of signal for 1 h followed by blocking with 5% milk in Tris-Tween-Buffer-Saline (TTBS buffer) for 1h. Primary antibody (1/1000) was added diluted in blocking solution for 1h at room temperature (RT) followed by 3 washes in TTBS. Secondary antibody (1/2500) in blocking solution was incubated 1h RT, washed and detected with Western Glo Chemiluminescent detection reagent.

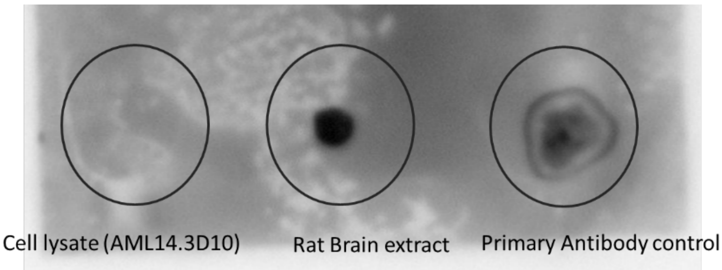

Figure S1. Dot Blot of mouse anti-human CRF in cell lysate (AML14.3D10 cells) and rat brain extract. This experiment confirms that no CRF is expressed in undifferentiated cells at baseline, while the positive sample tested and the primary antibody control show a clear signal.

2) CRF optimization of immune labelling in jejunal samples

As a control for signal to noise ratio in the protocol optimization, different dilutions of the secondary antibody (1/20; 1/50) and pH of incubation (7.4; 8.5) were tested. Unspecific signal was observed in mitochondria and in the nucleus of enterocytes, and in blood vessels (elements presumably lacking the antigen). Higher dilution of the secondary antibody (1/50) decreased this signal and higher pH conditions (pH 8.5) reduced background staining (figure S2).

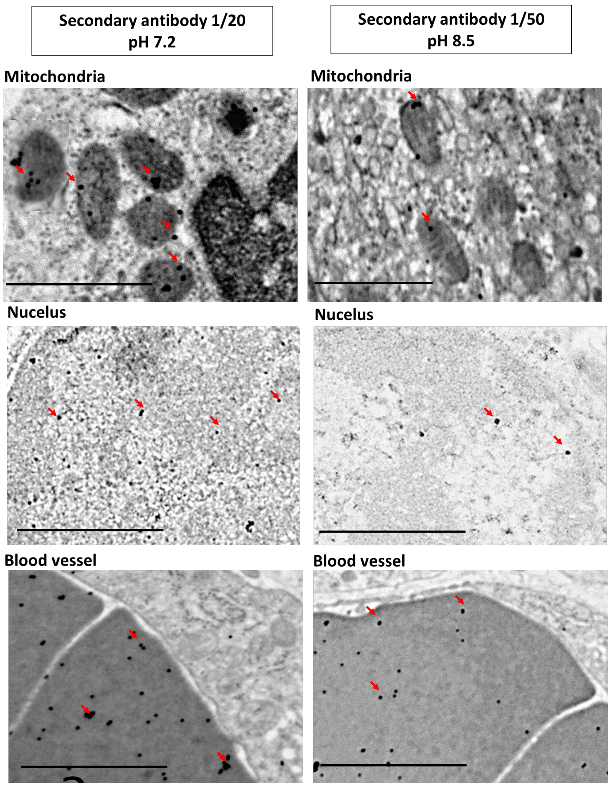

Figure S2. Representative micrographs showing unspecific labeling of the jejunal epithelium. Jejunal samples were incubated with the secondary antibody at different dilutions and pH. The unspecific signal observed in mitochondria and the nuclei of epithelial cells, and in erythrocytes within blood vessels were reduced increasing pH and dilution of the secondary antibody. Arrows indicate nanoparticles. Bars indicates 5μm.

The optimized conditions allowed a detailed ultrastructural analysis of all the lamina propria resident cells. Among mucosal leukocytes, positive CRF signal was detected only in eosinophil granules and negligible or absent in other immune cells (figure S3).

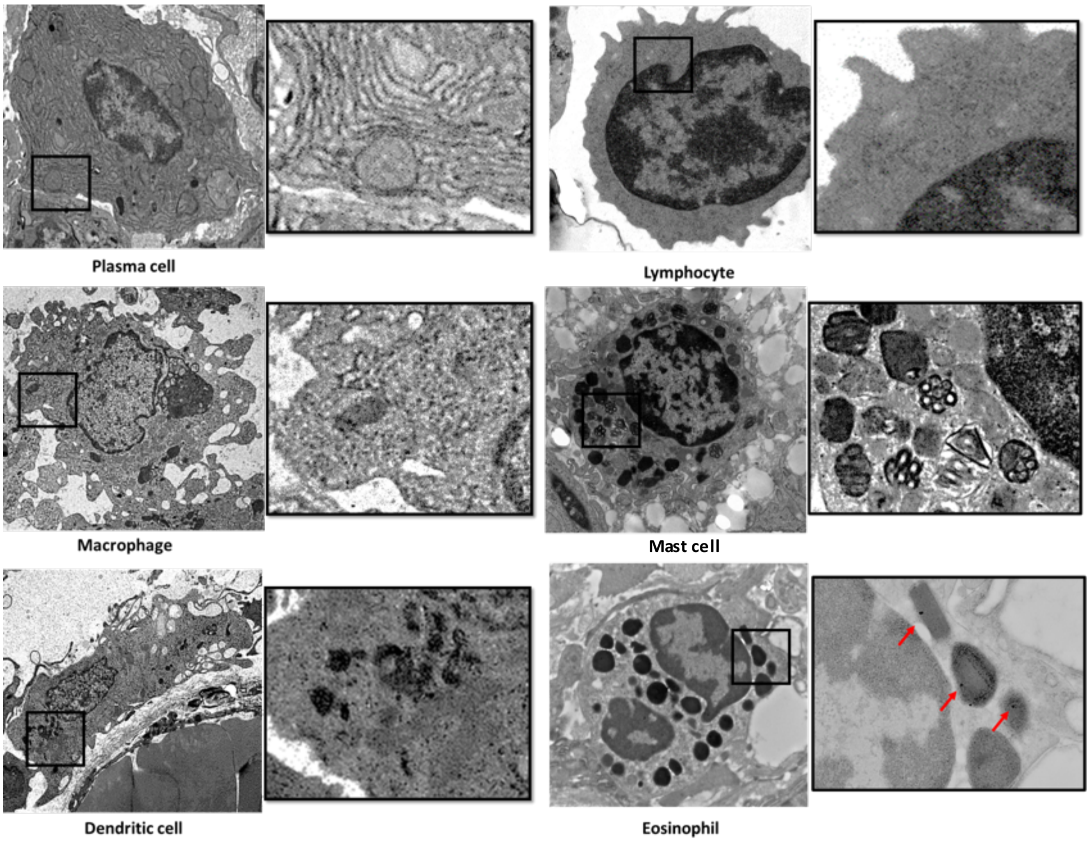

Figure S3. Specificity of CRF staining in eosinophils and not in other *lamina propria* resident cells in the jejunal mucosa. Representative micrographs of mucosal immune cells at different magnification. One representative plasma cell, lymphocyte, macrophage, mast cell, dendritic cell and eosinophil is shown. Arrows indicate CRF (visualized by nanoparticles) in eosinophil cytoplasmic granules of a healthy control sample (magnification 10.000x). Insert in each micrograph represents a detail of cell's cytoplasm in each cell type or granule (magnification 80.000x).

3) Isotype control of goat-anti-human CRF in eosinophil cell line 15HL-60

Goat anti-human CRF specificity in the eosinophilic cell line was confirmed by flow cytometry analysis (BD cytoperm protocol) by comparing positive signal versus negative and isotype control. The analysis of CRF signal (red), compared to negative and isotype control showed separation of CRF histogram (figure S4).

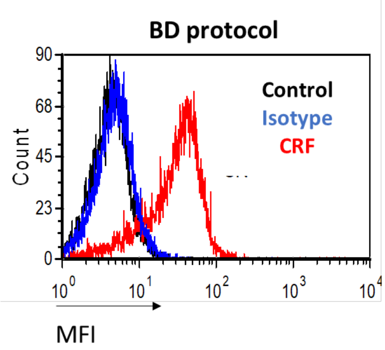

Figure S4. CRF and isotype control FACS histogram in 15HL-60 cells. Histograms showing CRF expression (MFI; x-axis) analyzed with specific goat anti-human CRF antibody (red curve) or isotype control (blue curve) or without primary antibody (black curve).

Eosinophil response to neuropeptides

The 15HL-60 cell line was maintained in culture in RPMI 1640 with 10% FBS, 1% penicillin-streptomycin and 1% L-Gln at pH 7.8. Cells were differentiated into mature eosinophils with 0.5 mM sodium butyrate for 7 days and stimulated with SP (10^{-6}M), CCh (10^{-4}M) or lipopolysaccharide (LPS) (1µg/mL) for 30, 90, 180 minutes and 24 hours. Antagonists (NK1R antagonist CP-96345 for SP; atropin for CCh) were added 30 min prior neuropeptide exposure at 10^{-6}M.

Flow cytometry immunostaining

Cell pellets ($2x10^5$ cells) were washed in cold PBS supplemented with 2% FBS and centrifuged at 300xg 7 min at 4ºC in round-bottom polystyrene tubes. Cell pellets were pre-treated with BD Cytofix/Cytoperm reagent (BD Biosciences) and incubated with primary antibody (table S3) for 1 hour on ice and with the secondary antibody (anti-goat IgG Alexa Fluor 488 conjugated) for 30 minutes. Cells were washed twice after every incubation and analyzed on FacsCalibur Flow Cytometer or LRSFortessa Flow Cytometer (BD Biosciences). Viability and apoptosis assay were performed in a separated experiment with and without stimulation and before and after permeabilization and staining.

Immunofluorescence analysis

Immunostaining was performed in suspension in round-bottom polystyrene tubes as described before for flow cytometry staining. A volume if 100 µL of stained cell suspension ($1x10^5$ cells) were added to a cytofunnel apparatus attached to a glass slide and centrifuged in a cytocentrifuge at 200xg for 6 minutes. Cytospin preparations were incubated for 10 min with DAPI (10ng/mL), washed twice with PBS and mounted with Prolong™ gold Antifade Mountant media (Invitrogen) and kept at 4ºC light protected until analysis.

B. Supplementary results:

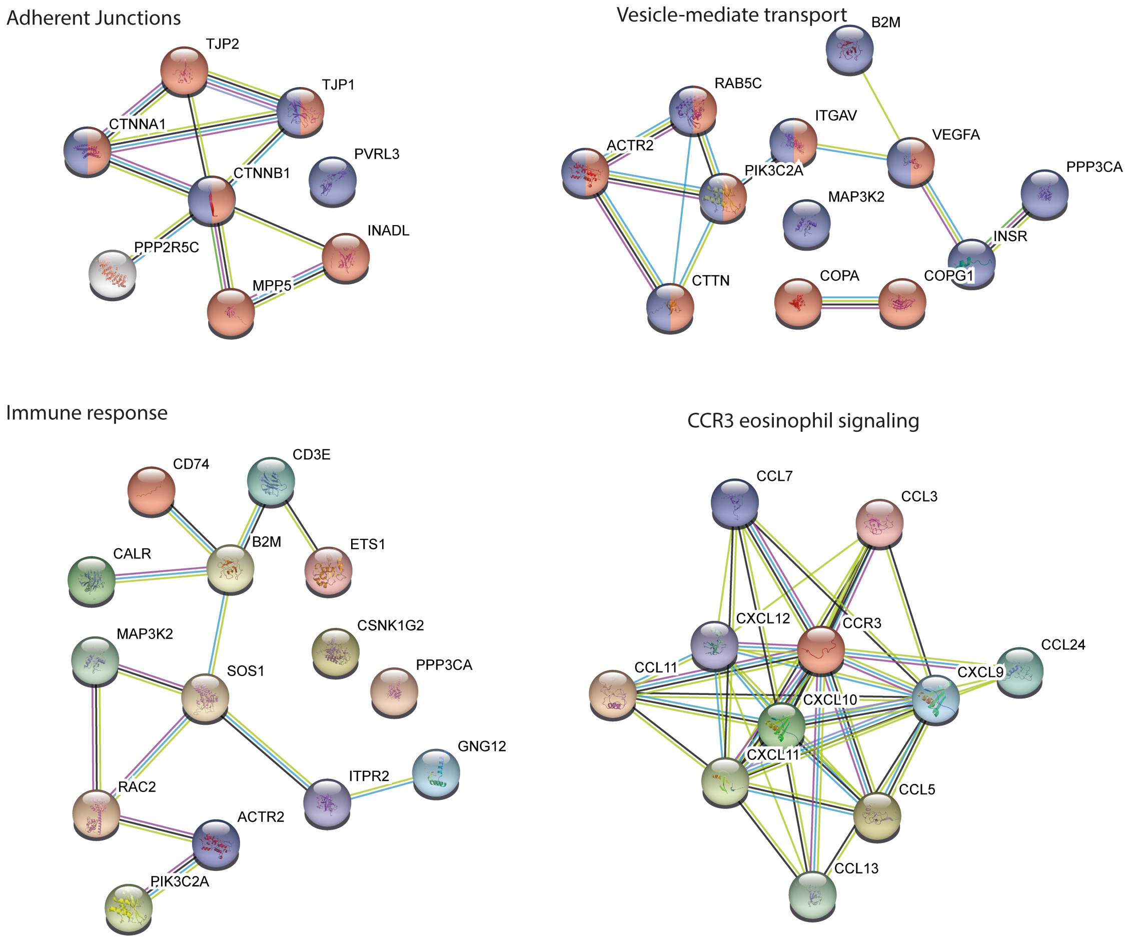

Figure S5. **Molecular networks of biological functions from jejunal transcriptome analysis.** Main molecular networks and associated biological functions (Adherent junctions, Vesicle-mediated transport, Immune response, CCR3 eosinophil signaling) obtained from the analysis of differentially-expressed transcriptome in IBS-D *vs*. HC in the jejunal mucosa by the IPA application for analysis, and then selected a list of genes and represented by molecular networks by https://string-db.org/.

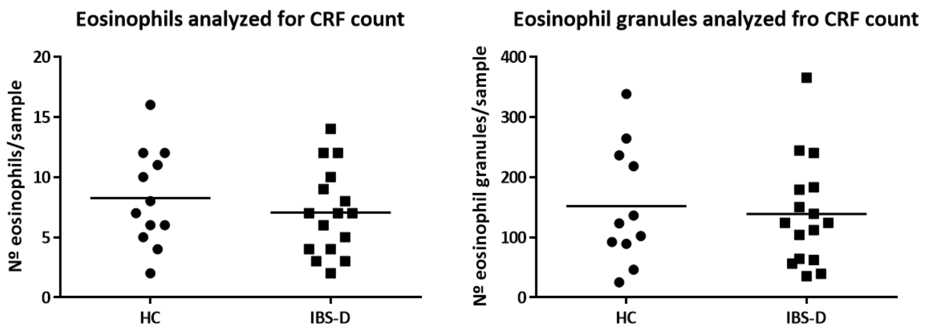

Figure S6. Analysis of eosinophils and cytoplasmic granules per group. Number of eosinophils and granules analyzed in HC and IBS-D subjects for CRF counting. CRF, corticotrophin releasing factor; HC, healthy control; IBS-D, diarrhea-predominant IBS.

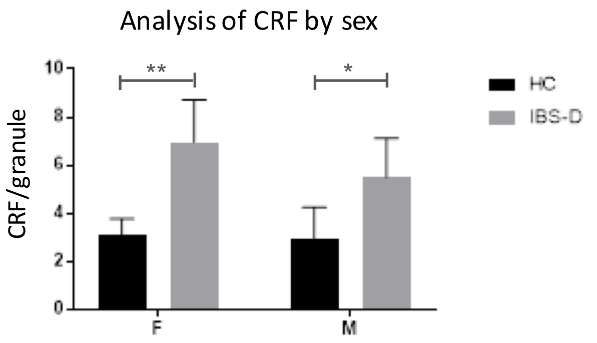

Figure S7. Analysis of mucosal eosinophil CRF content by sex. Number of CRF/granule in eosinophils analyzed by sex in HC and IBS-D patients. CRF, Corticotrophin releasing factor; F, female; HC, healthy control; IBS-D, diarrhea-predominant IBS; M, male. Statistical analysis was performed by two-way ANOVA followed by Mann-Whitney *U* test **P*<0.05 ***P*<0.001

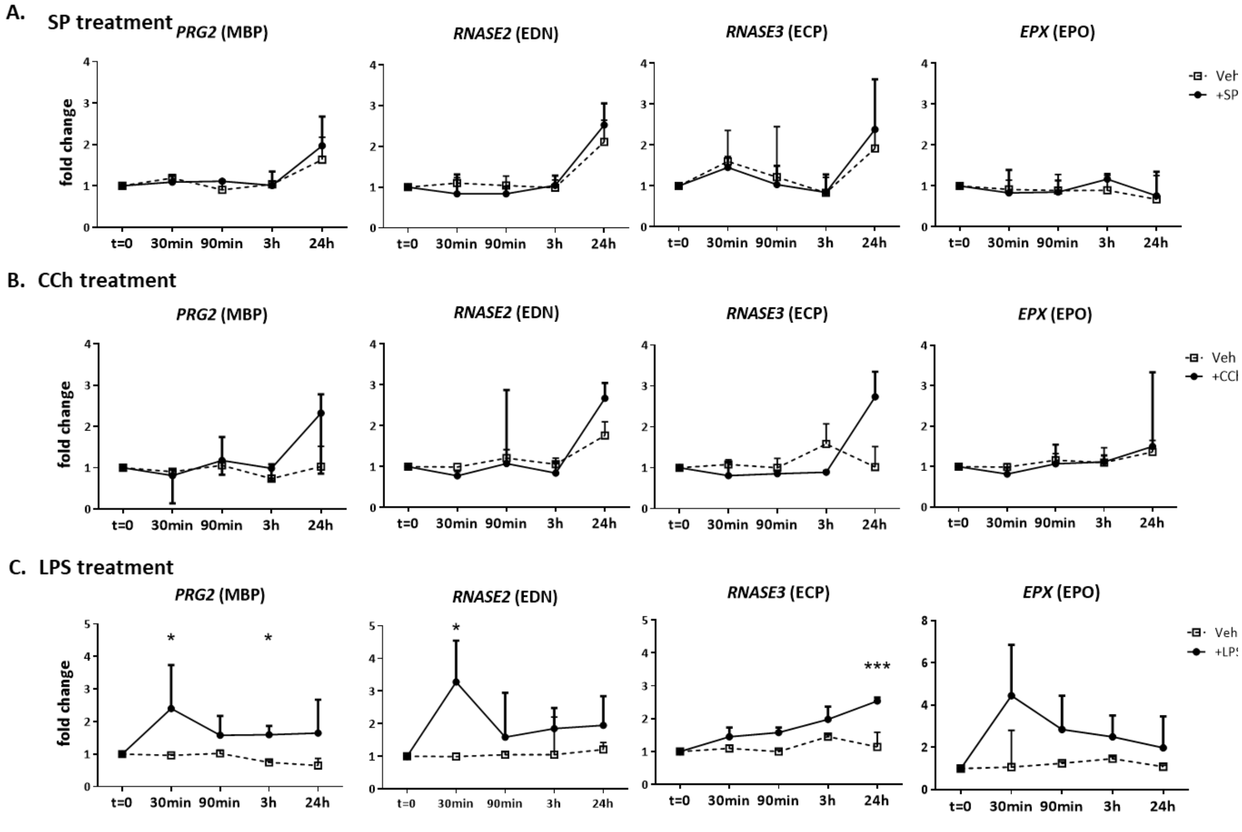

Figure S8. Time-course analysis of gene expression of eosinophil cationic proteins after stimulation with SP, CCh or LPS. Cells were treated with SP, CCh or LPS and vehicle control on the indicated time points and collected, and gene expression analyzed by qPCR. Fold-change was calculated by normalizing the data with the endogenous control gene (*PPIA*) and comparing each time point to untreated cells (t=0). Graphs represent results from 3 independent experiments. Data are expressed as median (range). Statistical analysis was performed by two-way ANOVA followed by Bonferroni multiple comparison post-hoc test **P*<0.05 ****P*<0.001 *vs.* vehicle. CCh: carbachol; LPS: lipopolysaccharide; SP: substance P; Veh: vehicle; *PRG2:* Proteoglycan 2; *RNASE2:* ribonuclease A family member 2; *RNASE3*: ribonuclease A family member 3: *EPX*: eosinophil peroxidase.
